# Analysis of the Patient Information Quality and Readability on Esophagogastroduodenoscopy (EGD) on the Internet

**DOI:** 10.1155/2018/2849390

**Published:** 2018-10-29

**Authors:** P. Priyanka, Yousaf B. Hadi, G. J. Reynolds

**Affiliations:** ^1^Department of Medicine, West Virginia University Hospitals, Morgantown, WV, USA; ^2^Department of Medicine, Section of Digestive diseases, West Virginia University Hospitals, Morgantown, WV, USA

## Abstract

**Objective:**

Patients are increasingly using the Internet to inform themselves of health-related topics and procedures, including EGD. We analyzed the quality of information and readability of websites after a search on 3 different search engines.

**Methods:**

We used an assessment tool for website quality analysis that we developed in addition to using validated instruments for website quality, Global Quality Score (GQS) and Health on Net (HON) certification. The readability was assessed using Flesch-Kincaid Reading Ease (FRE) and Flesch-Kincaid Grade level (FKG). 30 results of each search terms ‘EGD' and ‘Upper Endoscopy' from Google and 15 each from Bing and Yahoo were analyzed. A total of 45 websites were included from 100 URLs after removing duplicates, video links, and journal articles.

**Results:**

Only 3 websites were found to have good quality and comprehensive and authentic information. These websites were https://www.healthline.com, https://www.uptodate.com, and https://www.emedicine.medscape.com. There were additional 13 sites with moderate quality of information. The mean Flesch-Kincaid Reading Ease (FRE) score was 46.92 (range 81.6-6.5). The mean Flesch-Kincaid Grade level (FKG) was 11th grade, with a range of 6th grade to 12th grade and above making them difficult to read.

**Conclusions:**

Our study shows that there are quite a few websites with moderate quality content. We recommend 3 comprehensive and authentic websites out of 45 URLs analyzed for information on Internet for EGD. In addition, the readability of the websites was consistently at a higher level than recommended by AMA at 11th grade level. In addition, we identified 3 websites with moderate quality content written at 8th grade and below readability level. We feel that gastroenterologists can help their patients better understand this procedure by directing them to these comprehensive websites.

## 1. Introduction

There were 4.1 billion Internet users worldwide and 286 million within United States as of 2017, with 87.9% Americans having access to the Internet [1]. In one estimate, about 60% of the individuals with online access admitted going online to seek health-related information in 2013 [2]. This rapidly increasing use of web to seek information has made it possible for the patients to supplement their knowledge of medical conditions in a way that would not have been possible before the age of Internet. At the same time, the world wide web is still a largely unregulated place with a few rules to check the reliability or the accuracy of the information available. The content on the Internet is growing exponentially every year. This leads to the concern of either information overload where it is hard to determine relevant information from a barrage of sources or that patients may acquire information that might not be completely accurate and may affect the way they make important treatment decisions. A very few studies are available on the magnitude of this problem affecting gastroenterology patients seeking healthcare. The previously conducted studies on colorectal screening and non-GI conditions like knee arthroscopy, scoliosis, and ureteral stents have indicated that the online information available on these topics is highly variable in quality and mostly has suboptimal suitability and uniformly higher readability levels than AMA recommended 6th grade level for health information [3–8].

Esophagogastroduodenoscopy (EGD) is a widely performed gastrointestinal (GI) procedure since it first became available about a century ago [9]. In general, it is physicians' responsibility to explain the details of this procedure when it is warranted for either diagnostic or therapeutic purposes. But many times, patients turn towards Internet to get a better understanding of the various aspects of this procedure. About 6.9 million EGD procedures were performed in 2009 alone at an estimated cost of $12.3 billion [10]. A 50% increase in EGD utilization was noted among Medicare recipients from 2000 to 2010 and this trend continues to grow [11]. Currently, there is no exact information on the quality and readability of the web resources providing patient information on the topic of EGD. In this study, we tried to assess the quality and readability level of the online resources available to the patients on the topic of EGD. We also compared the results obtained from different search engines in an attempt to establish the most efficient search strategy.

## 2. Methods

### 2.1. Search Strategy

We used 3 different search engines for the purpose of this study, Google, Bing, and Yahoo. This was based on the popularity of the search engines with these three search engines cited to be among the most popular among the individuals seeking healthcare information [2]. The search terminology was “EGD” and “Upper Endoscopy” and typed as a phrase in each individual search engine. For the purpose of this study, we included the first 30 URLs from Google with each search term separately to obtain a total of 60 search results. We included first 15 URLs each from Bing and Yahoo with each search terminology. Overall, 100 search results were obtained and analyzed from these 3 different search engines. Of these 100 URLs, duplicates, video links, and research papers were excluded. Overall, 45 websites were selected for web resource quality and readability analysis.

### 2.2. Quality Assessment

The quality analysis was performed by using a comprehensive modified quality assessment questionnaire that was designed based on the methods used in previous similar studies (Table 1). Health on net (HON) certification and global quality score (GQS) were added to further refine the quality standards. HON Foundation is a nonprofit organization that grants certification to the websites with health-related information if they are in compliance with certain quality standards [12]. Each website was analyzed separately by 2 blinded observers using the above-mentioned questionnaire. Each item on the questionnaire was previously discussed and well-defined among the observers. For the adequacy of the content part, there were 6 subheadings and for each subheading the scores of 0, 3, and 5 could be given. Score of 0 indicated no information available on that subheading, 3 meant some information was available but suboptimal in content, and 5 was given if most of the information on that subheading was present. Similarly, authenticity scores of 0, 3, 5, and 10 were given if there were no references at all, website references, textbook references, or both textbook and scientific articles' references, respectively. HON certification, if present, was noted separately. GQS of 1-5 as mentioned in Table 1 was awarded separately by each observer. GQS has previously been used in similar studies to evaluate the overall quality and usefulness of a website [5, 13]. A final decision on the recommendation of a website was based on a score of at least 3 on all the subheadings under adequacy, at least 5 on authenticity, and a GQS of at least 4 and ideally had HON certification. We did not use HON certification as a final criterion for the recommending a website because only 3 websites we analyzed had HON certification and none of these 3 websites met our other quality criteria completely. For the items where the responses were different for each observer, a consensus was reached by discussion with the senior author, who was blinded with regard to the nature of the study. The mean interobserver reliability of the questionnaire was 0.94 (range 0.88-0.98). All the subcomponents of the quality assessment tool had interobserver reliability of >0.90 except GQS that had interobserver reliability of 0.88.

### 2.3. Readability Assessment

The readability of the websites was evaluated using Flesch-Kincaid Reading Ease (FRE) and Flesch-Kincaid grade level (FKG). FRE and FKG are widely used readability assessment tools validated for this purpose [14]. FRE is graded out of 100 and the easier text scores higher based on the sentence length and average number of syllables per word. The scores were calculated using Microsoft Word (Redmond, Washington) word processing software. The headings, web-links, illustrations, and foot notes were removed for the purpose of the readability assessment.

### 2.4. Statistical Analysis

Statistical analysis was performed using IBM SPSS software, version 22.0. Descriptive statistics were used for the quality and readability analysis of websites. Interobserver reliability was calculated to evaluate the quality of the questionnaire.

## 3. Results

### 3.1. Quality Analysis

Of 100 URLs, 45 were included in the final quality analysis. The remaining links were excluded as they were either video links, journal articles, PDF files, or duplicates. The search on Bing yielded 3 additional websites, and a search on Yahoo did not yield any unique website that was not previously identified on Google (Tables 2 and 3).

### 3.2. Information Update

The date of the most recent update of information was available only on 17 (38%) websites. Among these 17 sites, the median time since update was 14 months (range 0-76 months).

### 3.3. Content Presentation and Accessibility

All the 45 websites were easily accessible, except only 1 URL being inaccessible (page not found). None of the sites required user registration or were password protected. 15 of the 45 websites (33%) utilized illustrations or pictures to assist in the understanding of the procedure. Only 10 websites (22.2%) contained authorship information, with 9 of the 10 being either authored or reviewed by the physicians. Out of the 45 included websites, 20 (44.44%) contained promotional messages, 10 contained product related marketing messages, and 9 advertised for services. The target audience was recognized as the general public explicitly on 14 (31%) websites and no website identified its intended users as healthcare professionals. A total of 3 (6.6%) websites included in the final cohort were owned by the government agencies, 2 (4.4%) identified themselves as nonprofit, open access general information websites, 7 (15.5%) were for-profit strictly online resources, 3 (6.6%) were run by professional healthcare bodies, 13 (28.88%) were operated by educational healthcare institutions, and 15 (33.3%) were operated by private healthcare systems.

### 3.4. Content Quality Analysis

Out of the 45 websites analyzed, only 3 URLs were found to be adequate for the content per the predefined study criteria (Table 4). The rest of the 42 websites failed to satisfy the adequacy of content as criteria outlined previously. At least some mention of preprocedure, procedure-related, and postprocedure details was noted on 36 (91%), 41 (95%), and 38(84%) of the URLs. The complications were discussed only in 18 (40%), and the postprocedure warning signs were mentioned on 22 websites (48.9%). Only 5 (11%) websites had references available for the information presented and therefore could be considered authentic. HON certification was available only for 3 (7%) websites. Additionally, 13 more sites had a GQS > or equal to 4. Four websites were owned by professional bodies, 5 each were from educational institutions, private health systems, and for-profit online health information portals (Table 5). Of these, the search rank did not correlate with the chances of having better quality content.

### 3.5. Readability

The overall readability level of the websites was high, with mean Flesch-Kincaid Reading Ease (FRE) score of 46.92 (range 81.6-6.5). The mean Flesch-Kincaid grade level (FKG) was 11th grade, with a range of 6th grade to 12th grade and above. Only 2 websites had a reading grade level of 6 and below (medlineplus.gov, scripps.org) as recommended by AMA, and a total of 6 websites were written at the level of 8th grade and below (Table 6).

## 4. Discussion

In our study, we analyzed a sample of 100 web-links using 3 leading search engines. After the exclusion of the video links, journal articles, and repetitions 45 websites were identified to be included in our study for quality and readability analysis. Out of these 45 websites, only 3 were found to be recommendable, based on the adequacy criteria that comprised authenticity, content quality, and GQS (Table 2). Based on these results, our analysis shows that enormous amount of information is available regarding the EGD procedure on the Internet, mostly of moderate quality that may not be updated regularly. Although we intended to use HON as a criterion for website adequacy for recommendation, only 3 websites in our sample were found to have HON certification, and while all three had a GQS of 4, they were found to be deficient in one or more content quality subcomponents and could not be included in the final list of recommendable websites. Only less than one-third of the sites had clearly identified target audience as patients and less than a quarter websites had authorship information available, prompting a concern about the source of information about the rest of three-quarters of the content. About half of the sites included in this study were using their website for promotional messages or advertisements that may lead to potential conflicts of interest and undermine their seriousness about the patients' well-being. After the subheading analysis of content quality analysis, although most websites discussed indications, preprocedure, procedure, and postprocedure somewhat adequately (80-95%), only about less than half mentioned the possible complications of the procedure (40%) and warning signs to recognize them (48.9%). This pattern was noted for both for-profit and nonprofit websites like educational institutions and government owned websites, though it was seen more frequently with the privately owned websites. This trend is worrisome as these websites seemed to make patients aware of the procedure without educating them adequately of the associated risks and even worse, to recognize the complications if they occurred. This also speaks somewhat about us as a medical community where we sometimes underinform our patients of the possible risks of the procedures in a subconscious attempt to not scare patients by discussing the complications in detail. 13 websites with GQS of at least 4 that did not fulfill all the quality criteria could still be considered as reliable with at least moderate quality content (Table 5). Not surprisingly, most of these websites were owned by nonprofit organizations like professional bodies, government, and educational institutions.

For the readability analysis, the median FRE score was 46.92, consistent with an 11th grade reading level. None of these websites were determined to be having adequate content per our quality criteria. The two websites written at the 6th grade level were both HON certified but failed to meet our adequacy criteria due to absent information in one or two subcategories. These findings emphasize the challenges faced by the low education achievement patients seeking good quality information presented in a manner appropriate for their reading skills. We were able to recognize at least 3 websites with readability level of 8th grade or below and GQS of 4 in an attempt to help this cohort of patients. (Table 6)

It can be safely assumed that the trend of using the Internet is going to be ever expanding in the medical decision-making for many of our patients. The use of Internet by the patients has been a topic of debate in various medical and surgical specialties. As early as 1997, a study reviewed the websites on the cancer treatments in an attempt to recommend those sites to the patients [15]. A few other studies have examined the quality and readability of the topic specific information on the world wide web [4–7, 16]. In a study in 2001 on online information on intersex anomalies, 6 different general search engines were used and first 50 search results were included [16]. They concluded that of the 300 websites analyzed, only 45 were found to have patient related information and only 5 were recommendable (1.6%). This was similar to our study, where we used 3 different search engines with 100 website links and 45 were analyzed and 3 were found to have high-quality information but none of these having readability levels of 8th grade and below. Similarly, John et al. in 2016 analyzed 80 articles using different search terms for colorectal cancer screening including colonoscopy, flexible sigmoidoscopy, fecal occult blood test and CT colonography for the readability and overall quality [4]. Similar to our study results, they found that these 80 sites were written at 11.7 grade level in contrast to the recommended 3rd to 7th grade levels by AMA and NIH. This study also found reliability, accessibility, and usability of these websites to be moderate.

We did not find false or misleading information on EGD in the web pages that we searched. No portals or discussion forums were encountered among the search results obtained using our search strategy. Therefore, there was a general lack of subjectivity in the web pages that were obtained. EGD is a commonly performed procedure and is likely to be searched more than other GI procedures except perhaps colonoscopy. The conclusions from this study regarding the quality of information available on the Internet for EGD, therefore, cannot be extrapolated for other GI procedures.

It remains to be studied, however, if the patients prefer to use other applications like social media including Twitter, Reddit, and Facebook as important resources for health information. Either large organizations or healthcare institutions operated most of the web sites that were included in our analysis. While the search engines like Google and Bing have developed complex algorithms, and the web pages that are suggested to users appear in a sequence that is in part generated by the relevance and authenticity of the web site, searches on social media may be more liable to subjective opinion. This concern has recently been studied by Stock et al., who found while studying cleft lip and palate that although social media groups provided an avenue for real-time health discussion and were frequently used, they suffered from the disadvantage of reliance on opinion and subjective experience [17]. Regardless, as a growing avenue for obtaining health information on the Internet, this aspect of the world wide web needs further investigation.

Our study highlights the challenges faced by the patients in successfully navigating the Internet when making important healthcare decisions involving the use of EGD. Our analysis shows that most of the information available online is moderate quality with some comprehensive and reliable websites, but it can be difficult to find these resources and cause confusion to the readers. This puts gastroenterologists in a unique situation where we need to encourage our patients to make informed decisions and balance it with the information available online. We believe gastroenterologists should be more aware of the quality of the resources available on the Internet for EGD and other procedures to provide better patient experience. We feel that the role of physicians here could be in directing the patients to high-quality websites to supplement their knowledge of the EGD procedure. We envision that physicians should be able to use these resources to facilitate the thorough understanding of the procedure and make informed decisions when patients elect to have EGD. This may require closing the loop of communication with the patients by encouraging patients to get back to the physicians after they had a chance to go through these high-quality recommendable websites.

The strengths of our study are that we have targeted an extremely common GI procedure for which no current data on the quality of online resources exists in the scientific literature. We used multiple search engines in an attempt to come up with the best search strategy on this topic. Our study showed that there was not much added benefit to using different search engines for obtaining the high-quality results. Another unique feature of our study was that we were able to identify 3 overall good quality content websites and another 3 websites for lower readability level patients to better assist them in understanding this procedure.

We recognize that our study had some limitations as well. We are aware that the order of the search results obtained by the individual patients may not be strictly the same as those obtained by us due to geographical location variations, previous search history, and cookies on individual computers. We are also cognizant of the dynamic nature of the Internet and the fact that this study was cross-sectional in design. Our search was limited to English language results and there are many users on the Internet who prefer languages other than English and the results of this study may not be applicable to these patients.

## 5. Conclusions

Our study shows that there is a wide variation in the content of the websites available on EGD on the Internet. There are quite a few websites with moderate quality content but authenticity of the content remains a challenge. We could analyze 3 comprehensive and authentic websites out of 45 URLs and 13 other moderate quality websites. In addition, the readability of the websites was consistently at higher level than recommended by AMA. We identified 3 websites with moderate quality content written at 8th grade and below readability level. We feel that the active involvement of gastroenterologists in directing their patients to superior information quality websites will help their patients understand the EGD procedure better and help prevent miscommunication regarding its nature and risks.

## Figures and Tables

**Table 1:** Assessment tool for the website quality analysis.

**Search Engine **Google ** ** Bing ** ** Yahoo
**Website description**
URL address
Type of ownership
Position in search result
Accessibility ** ** Easy ** ** Page not found ** ** No longer exists ** ** Password-protected
Illustrations and pictures Y / N
**Quality **
Last information update Y / N If yes, how old?
Authorship information available, Y/ N If yes, easy to find Y / N
If yes, is author identified as - General Public, Educational institution, Club,
Prof organization, For-profit organization., physician.
Promotional message Y / N
What is being promoted? Product / service / advertisement / procedure
Target audience information Y/N
Type of target audience General public / HCPs
Adequacy of content (0, No information, 3, Some information, 5, adequate information)
Indications 0 3 5
Pre-procedure preparation 0 3 5
Procedure 0 3 5
Post procedure protocol 0 3 5
Complications 0 3 5
Warning signs of complications 0 3 5
Total
Authenticity of the content 0 3 5 10
0-No referencing at all, 3-Good quality website referenced, 5-textbook referenced
10-Textbook and scientific articles referenced
**HON certification** Yes / No
**Global Quality Score**: ___
1 Poor quality, poor flow of the site, most information missing, not at all useful for patients.
2 Generally poor quality and poor flow, some information listed but many important topics missing, of very limited use to patients.
3 Moderate quality, suboptimal flow, some important information is discussed adequately but other information is poorly discussed,
somewhat useful for patients.
4 Good quality and generally good flow, most of the relevant information is listed, but some topics are not covered, useful for patients.
5 Excellent quality and excellent flow, very useful for patients.
Would you recommend the site Y / N
**Readability:** FRE: ___ FKS grade level: ___

**Table 2:** Search results with website URLs.

** Website urls**	**Website number**
https://medlineplus.gov/ency/article/003888.htm	1
https://www.healthline.com/health/egd-esophagogastroduodenoscopy	2
http://ddc.musc.edu/public/procedures/upper-endoscopy.html	3
https://en.wikipedia.org/wiki/Esophagogastroduodenoscopy	4
https://www.hopkinsmedicine.org/gastroenterology_hepatology/clinical_services/basic_endoscopy/esophagogastroduodenoscopy.html	5
https://www.cancercenter.com/treatments/esophagogastroduodenoscopy/	6
https://www.webmd.com/digestive-disorders/upper-endoscopy#1	7
https://www.scripps.org/articles/273-egd-esophagogastroduodenoscopyhttps://www.scripps.org/articles/273-egd-esophagogastroduodenoscopy	8
https://www.northshore.org/gastroenterology/procedures/egd-test/	9
https://emedicine.medscape.com/article/1851864-overview	10
http://ohiogi.com/procedurespreps/procedures/upper-endoscopy-esophagogastroduodenoscopy-egd/	11
https://www.valleyhealth.com/gastrointestinal_services.aspx?id=2690	12
https://www.aurorahealthcare.org/services/gastroenterology-colorectal-surgery/esophagogastroduodenoscopy	13
https://www.uofmhealth.org/conditions-treatments/digestive-and-liver-health/upper-endoscopy-egd	14
https://medicine.yale.edu/intmed/digestivediseases/clinical/Egd%20STENT%20pt%20handout%207.17_270191_1095_23162_v2.pdf	15
https://www.gihealthcare.com/egd/	16
https://www.gastrorockies.com/preps/colonoscopy-egd-prep-instructions	17
http://gastroarkansas.com/egd-esophagogastroduodenoscopy/	18
http://www.arizonadigestivehealth.com/procedures-services/upper-gi-endoscopy/	19
http://www.riverviewmedicalcenter.com/RMC/services/Endoscopy/UpperEndoscopy.cfm	20
https://www.michigangastro.com/upper-gi	21
https://www.drugs.com/mcp/upper-endoscopy	22
https://medical-dictionary.thefreedictionary.com/EGD	23
http://www.gastroenterology.com/procedures/egd	24
https://www.mayoclinic.org/tests-procedures/endoscopy/about/pac-20395197	25
https://www.asge.org/home/for-patients/patient-information/understanding-upper-endoscopy	26
https://www.niddk.nih.gov/health-information/diagnostic-tests/upper-gi-endoscopy	27
https://www.cancer.net/navigating-cancer-care/diagnosing-cancer/tests-and-procedures/upper-endoscopy	28
https://www.medicinenet.com/endoscopy/article.htm	29
https://www.uptodate.com/contents/upper-endoscopy-beyond-the-basics	30
https://www.sages.org/publications/patient-information/patient-information-for-upper-endoscopy-from-sages/	31
https://www.gastro.org/practice-guidance/patientInfo/procedures	32
http://www.jerseyshoreuniversitymedicalcenter.com/JSUMC/services/gastroenterology/UpperEndoscopy.cfm	33
https://stanfordhealthcare.org/medical-conditions/cancer/stomach-cancer/stomach-cancer-diagnosis/upper-endoscopy.html	34
http://www.mountsinai.org/patient-care/service-areas/digestive-disease/endoscopy-suite/types-of-endoscopy-procedures/egd-or-upper-endoscopy	35
https://www.mskcc.org/cancer-care/patient-education/about-your-upper-endoscopy	36
https://www.gikids.org/content/59/en/endoscopy/upper	37
http://www.chp.edu/our-services/transplant/intestine/education/patient-procedures/upper-endoscopy	38
https://www.cincinnatichildrens.org/health/u/upper-endoscopy	39
https://www.cancer.gov/publications/dictionaries/cancer-terms/def/upper-endoscopy	40
http://www.morethanheartburn.com/testsandtreatments/upper-endoscopy	41
http://health.usf.edu/medicine/internalmedicine/digestive/upperendoscopy	42
http://www.gandhofcny.com/procedures/upper-endoscopy/	43
https://www.gastro.org/practice-guidance/patientInfo/procedures	44

**Table 3:** Results of website content analysis and readability assessment.

**Website number**	**Adequacy total**	**Authenticity**	**Overall content **	**Recommended**	**FRE**	**FKS**	**HON**	**GQS**
1	21	10	good	yes	81.6	6	yes	5
2	30	10	good	yes	57.3	9	no	5
3	25	10	good	yes	58.6	9	no	4
4	19	3	fair	no	49.3	10	no	3
5	16	0	fair	no	51	9.3	no	3
6	9	0	fair	no	46.6	12	no	3
7	30	0	good	yes	57.4	9	no	5
8	16	0	fair	no	74.4	6	yes	4
9	16	0	fair	no	57.5	9	no	3
10	30	10	good	yes	-6.5	12	no	5
11	30	0	good	yes	46	11	no	5
12	18	0	fair	no	46.2	12	no	3
13	9	0	poor	no	63.3	8	no	2
14	26	0	good	yes	46.9	11	no	4
15	23	0	fair	no	57.1	10	no	3
16	18	0	fair	no	48.7	12	no	3
17	15	0	poor	no	59.7	10	no	3
18	14	0	poor	no	50.5	11	no	3
19	25	0	good	yes	33.6	12	no	4
20	21	0	fair	no	44.6	11	no	4
21	30	0	good	yes	26.9	12	no	5
22	30	0	good	yes	36.4	13	yes	5
23	15	0	poor	no	35.9	13	no	2
24	16	0	fair	no	46	12	no	3
25	30	0	good	yes	38.2	12	no	5
26	24	0	fair	yes	37.4	12	no	4
27	28	0	fair	yes	29.6	13.2	no	4
28	18	0	fair	no	56	8	no	4
29	15	0	fair	no	21	16.3	no	3
30	30	10	good	yes	38.2	13.3	no	5
31	26	0	good	yes	35	12.1	no	5
32	18	0	fair	no	38.9	13	no	3
33	3	0	poor	no	61.2	8	no	2
34	26	0	good	yes	37.1	15.6	no	5
35	16	0	fair	no	58	10	no	3
36	17	0	fair	no	43	12.73	no	3
37	19	0	fair	no	50	11	no	3
38	6	0	poor	no	50	10	no	2
39	6	0	poor	no	64	8	no	2
40	14	0	fair	no	43	12	no	3
41	17	0	fair	no	45	12	no	3
42	11	0	fair	no	61	8	no	3
43	23	0	good	no	48	10	no	4
44	PNF	PNF	PNF	PNF	PNF	PNF	PNF	PNF

GQS: global quality score, HON: health on net certification, FRE: Flesch-Kincaid Reading Ease (FRE), FKG: Flesch-Kincaid grade level, and PNF: page not found.

**Table 4:** Websites found to have adequate content on EGD.

https://www.healthline.com/health/egd-esophagogastroduodenoscopy
https://emedicine.medscape.com/article/1851864-overview
https://www.uptodate.com/contents/upper-endoscopy-beyond-the-basics

**Table 5:** Websites with moderate quality content on EGD (GQS of 4 or more).

https://medlineplus.gov/ency/article/003888.htm
http://ddc.musc.edu/public/procedures/upper-endoscopy.html
https://www.webmd.com/digestive-disorders/upper-endoscopy#1
http://ohiogi.com/procedurespreps/procedures/upper-endoscopy-esophagogastroduodenoscopy-egd/
https://www.uofmhealth.org/conditions-treatments/digestive-and-liver-health/upper-endoscopy-egd
http://www.arizonadigestivehealth.com/procedures-services/upper-gi-endoscopy/
https://www.michigangastro.com/upper-gi
https://www.drugs.com/mcp/upper-endoscopy
https://www.mayoclinic.org/tests-procedures/endoscopy/about/pac-20395197
https://www.asge.org/home/for-patients/patient-information/understanding-upper-endoscopy
https://www.niddk.nih.gov/health-information/diagnostic-tests/upper-gi-endoscopy
https://www.sages.org/publications/patient-information/patient-information-for-upper-endoscopy-from-sages/
https://stanfordhealthcare.org/medical-conditions/cancer/stomach-cancer/stomach-cancer-diagnosis/upper-endoscopy.html

**Table 6:** Websites with moderate quality content and readability level of 8th grade and less.

https://medlineplus.gov/ency/article/003888.htm
https://www.scripps.org/articles/273-egd-esophagogastroduodenoscopy
https://www.cancer.net/navigating-cancer-care/diagnosing-cancer/tests-and-procedures/upper-endoscopy

## Data Availability

The data used to support the findings of this study are available from the corresponding author upon request.
